# Knowledge, Attitude and Practices Toward Coronavirus Disease (COVID- 19) in Southeast and South Asia: A Mixed Study Design Approach

**DOI:** 10.3389/fpubh.2022.875727

**Published:** 2022-06-21

**Authors:** Mohammad Meshbahur Rahman, Roy Rillera Marzo, Shanjida Chowdhury, Sikandar Ali Qalati, Mohammad Nayeem Hasan, Gowranga Kumar Paul, Khadijah Abid, Wegayehu Enbeyle Sheferaw, Angela Mariadass, Divitra Chandran, Shasvini Kanan, Ahmad Umar Shafie Bin Ahmad Firdaus, Fatimah Az Zahra' binti Sabarin, Yulan Lin

**Affiliations:** ^1^Department of Biostatistics, National Institute of Preventive and Social Medicine, Dhaka, Bangladesh; ^2^Department of Community Medicine, International Medical School, Management and Science University, Selangor, Malaysia; ^3^Department of Community Medicine, Faculty of Medicine, Asia Metropolitan University, Johor, Malaysia; ^4^Global Public Health, Jeffrey Cheah School of Medicine and Health Sciences, Monash University Malaysia, Subang Jaya, Malaysia; ^5^Department of General Educational Development, Daffodil International University, Dhaka, Bangladesh; ^6^School of Finance and Economics, Jiangsu University, Zhenjiang, China; ^7^Department of Statistics, Shahjalal University of Science and Technology, Sylhet, Bangladesh; ^8^Department of Statistics, Mawlana Bhashani Science and Technology University, Santosh, Bangladesh; ^9^Department of Public Health, The Shaheed Zulfikar Ali Bhutto Institute of Science and Technology, Karachi, Pakistan; ^10^Department of Statistics, Mizan-Tepi University, Tepi, Ethiopia; ^11^Department of Epidemiology and Health Statistics, School of Public Health, Fujian Medical University, Fuzhou, China

**Keywords:** COVID–19, knowledge, attitude, practice, Southeast and South Asia

## Abstract

**Background:**

Coronavirus has spread to almost every country since its emergence in Wuhan, China and countries have been adopted an array of measures to control the rapid spread of the epidemic. Here, we aimed to assess the person's knowledge, attitude and practices (KAP) toward the COVID-19 epidemic in Southeast and South Asia applying the mixed study design (cross-sectional and systematic review).

**Methods:**

In the cross-sectional study, 743 respondents' socio-demographic and KAP-related information was collected through an online population-based survey from the Malaysian population. In the systematic review, the database PubMed, Web of Science and Google Scholar search engine were searched and related published articles from South and Southeast Asia were included. Frequency distribution, Chi-square association test and binary logistic regression were fitted using cross-sectional data whereas random effect model and study bias were performed in meta-analysis. We used 95% confidence interval and P <0.05 as statistical significances.

**Results:**

The prevalence of good knowledge, positive attitude and frequent practice toward COVID-19 epidemic were 52.6%, 51.8% and 57.1%, respectively, obtained by cross-sectional data analysis. The KAP prevalence were ranged from 26.53% (Thailand) to 95.4% (Nepal); 59.3% (Turkey) to 92.5% (Pakistan); and 50.2 (Turkey) to 97% (Afghanistan), respectively, obtained by 18 studies included in the meta-analysis. The prevalence of KAP was higher [84% vs. 79%, *P*_*heterogeneity*_ <0.001; 83% vs. 80%, *P*_*heterogeneity*_ <0.001; 85% vs. 83%, *P*_*heterogeneity*_ <0.001] in South Asia compared to Southeast Asia, obtained by subgroup analysis. Some studies reported mean level instead of the proportion of the KAP where the score varied from 8.15–13.14; 2.33–33.0; and 1.97–31.03, respectively. Having more knowledge and attitude were encouraged more likely to practice toward COVID-19. Study suggests age, gender, education, place of residence and occupation as the most frequent significant risk factors of KAP toward COVID-19.

**Conclusion:**

The study sufficiently informs how other countries in Southeast and South Asia enriches their KAP behaviors during the pandemic which may help health professionals and policymakers to develop targeted interventions and effective practices.

## Introduction

On 11th March 2020, the World Health Organization [WHO] declared coronavirus (COVID-19) a global pandemic (1). The virus has spread to almost every country since its emergence in Wuhan, China (2–5). As of 12 April 2022, worldwide, 497.96 million cases and 6.18 million deaths have been confirmed, whereas 11,250.78 million vaccine doses have been administered globally, reported by the WHO (6). In Asia and Southeast Asia, over 71.96 million and 37.34 million confirmed cases have been reported respectively (7). On 25th January 2020, Malaysia reported the first coronavirus case (8) and the first two COVID-19 deaths were confirmed on March 17, 2020 by the Malaysian government (9). On 11th September 2021, Malaysia has experienced 1.92 million positive cases, including 19,486 deaths (10).

The WHO suggested various strategies and measures such as social distancing, using sanitizers and wearing masks, regular hand washing, avoid visiting crowded places and lockdowns to control the spread of the disease (11–13). Mass vaccination has also been applied widely to beat the deadly pandemic, and the hesitancy in populations have raised severe concerns (14–18). Although, the lockdowns decision was not easy for the developing counties as it affected people and countries' economic performance very badly. In a study of Fernades (2020), he reported that about 10–15% decrease in the GDP of 30 countries (19). However, in order to mitigate the large-scale spread of COVID-19 and to reduce the pressure on the country's health facilities, the Government of Malaysia launched the “Movement Control Order (MCO)” on 18th March 2020 (1). Same as the lockdowns in other Southeast and South-Asian countries, the MCO prohibited unwanted movements outside the home, prohibited citizens from traveling and also prohibited the entry of all foreigners. Individuals were allowed to leave the house only for necessary needs such as medical care and grocery purchase. All industries which were not necessary were ordered to close or enable employees to work from their own homes (1, 5, 20).

The success or failure of all the efforts made by MCO was directly depends on the public's way of adopting the standard operating procedures (SOPs) set by the government to control the spread of COVID-19. When MCO declared its first announcement, many people were confused and panicked (20). In addition, some people were traveling crowded hubs and return to their hometowns and as a consequence, the chances of virus spreading were also increasing. Therefore, this public response to MCO were not been anticipated and poses concerns about individual's degree of knowledge and attitudes toward COVID-19 (21). In the Asian developing countries, the scenario was worse effective. In a study, Qalati et al. (22) reported that the effectiveness of lockdown is based on the cooperation and compliance of society members (22). Furthermore, another online survey conducted during the MCO period reported that 64% of Malaysians had good awareness toward COVID-19 prevention, while 65% of individuals had positive attitude and 57% had adequate practice, respectively. This survey also showed that low education level was the significant risk factor for knowledge, attitude and practice (KAP) regarding COVID-19 prevention among Malaysians (21). Another small-scale study reported that older age, illiterate, rural resident and lower income group were more vulnerable to KAP whereas, the positive attitude toward controlling of virus was significantly different among age groups, occupation and religion. Furthermore, most of the respondents reported they properly wash their hands and use hand sanitizer (88%) as a precautionary measure (1).

A study in the Philippines conducted earlier stages of the pandemic showed that 94.0% of respondents had already heard of COVID-19 and their main sources of knowledge are television and radio. A big percentage think that coughing and sneezing are the main transmission route and hand washing are also a preventive measure against the virus. But they are not aware about social distancing (23, 24). Another study in Indonesia showed that over two-thirds of respondents correctly answered questions related to COVID-19 general symptoms, transmission modes, and prevention measures. Most of the respondents agreed that COVID-19 might be controlled and Indonesia might win against this virus (25).

The risk factors associated with KAP toward COVID-19 is necessary to investigate in order to specify the target groups where interventions are needed for behavioral change. There is currently a little information available on the KAP in a complete form in Southeast and South Asia. In addition, research in mixed study design is a limited approach devoted to COVID-19 so far in the context Southeast and South-Asian countries. Most of the previously published studies are cross-sectional or review based. But a researcher can get a complete and more sufficient assessment about a phenomenon by performing a mixed study design research. Assessing the risk factors linked to KAP toward COVID-19 would aid in generating data that could be used to develop tailored strategies and health promotion initiatives. Therefore, this study initiated a mixed study design (cross-sectional study with systematic review and meta-analysis) to assess the knowledge, attitude and practices toward the COVID-19 epidemic in Southeast and South Asia.

## Methods

This study was conducted by following a mixed study design (cross-sectional and systematic review). For this, we firstly performed an online-based cross-sectional study among the Malaysian population. Later, we performed a systematic review to obtain a comprehensive scenario on KAP in Southeast and South Asia.

### Methods Used in Cross-Sectional Study

#### Data and Study Population

In the cross-sectional study, a sample of 743 respondents' information was collected through an online population-based survey. The criteria for including participants were willing to participate, currently being a Malaysian resident, having access to the internet. Information involving the person-centered knowledge, attitude and practices assessment toward COVID-19 were collected using a formal Google based questionnaire.

#### Data Collection Procedures

We distributed the questionnaire using personal contacts by emails, web-based applications (e.g., WhatsApp, Telegram), and social media (e.g., Facebook, LinkedIn, Twitter, and Instagram). Participants must be aged 18 years or older. They were reminded to respond only once and use a unique identifier to create a single account by settings that allow one response per user. Finally, the confidentiality and privacy of participants' responses were ensured to minimize potential bias caused by self-reported data.

Data collected using google forms offered an excel format to survey output to analyze the raw data offline. All country's data in excel were exported to IBM SPSS (version 25). De-identifications were done to secure the safety of respondents, which removed the identifiers like name, email ID, or mobile number that directly or indirectly point to a person.

#### Study Questionnaire/Tool

Sociodemographic characteristics of the participants included age, gender, residence, ethnicity, nationality, education level, occupation, marital status, number of family members and family income.

Respondents' knowledge, attitude and practices toward COVID-19 were the three outcome variables of the study. The knowledge section consisted of 10 questions and each question had a possible response of “Yes” and “No”. The correct answer (Yes) was coded as 1, while the wrong answer (No) was coded as 0. The total score ranged from 0–10, with an overall greater score indicates more accurate knowledge. A cut of the level of ≥7 was set for more accurate knowledge in the study, which was the Median value of the distribution of knowledge score (26).

The attitude section also consisted of 10 items and the response of each item was indicated on a 4-point Likert scale as follows 1 (“Strongly disagree”), 2 (“Disagree”), 3(“Agree”), and 4 (“Strongly Agree”). The total score was calculated by summating the ten questions' raw scores ranging from 10 to 40, with an overall greater score indicating more positive attitudes toward COVID-19. A cut-off level of ≥33 (median value) was set for more positive attitudes toward preventing COVID-19 (27).

The practice section also included 10 items of practice measures responding to the COVID-19, and each item was answered 1(“Never”), 2(“Sometimes”), 3(“Often”), and 4(“Very often”). Practice items' total score ranges from 10–40, with an overall greater score indicates more frequent practices toward the COVID-19. A cut-off level of ≥33 (median value) was set for more frequent practices. For all the cases, coding is evident (27).

#### Data Reliability and Validity

To confirm the data reliability and validity, we used several techniques: (i) checked the data response error. (ii) explored data with graphical representation. (iii) performed reliability analyses of the data (Cronbach's alpha coefficient for knowledge, attitude, and practice were 0.38, 0.79, and 0.82, respectively, and overall alpha coefficient of KAP questions was 0.81); and (iv) use of two statistical techniques, namely chi-square test and logistic regression (28, 29).

### Methods for Systematic Review and Meta-Analysis

The Preferred Reporting Items for Systematic reviews and Meta-Analyses (PRISMA)-2020 guideline (Supplementary Table S1) was followed in the systematic review and meta-analysis part of our study (30).

#### Search Strategy

In the systematic review part, the database PubMed, Web of Science and Google Scholar search engine were searched, and related published articles during 2019–2020 from South and Southeast Asia were screened. The keywords used in the database search were “prevalence”, “proportion,” “risk factors,” “knowledge,” “awareness,” “practice,” and “knowledge on COVID-19,” “attitude on COVID-19” and “learning on COVID-19”. We also manually scanned the reference lists of the included articles.

#### Inclusion and Exclusion Criteria

**Figure 1:**
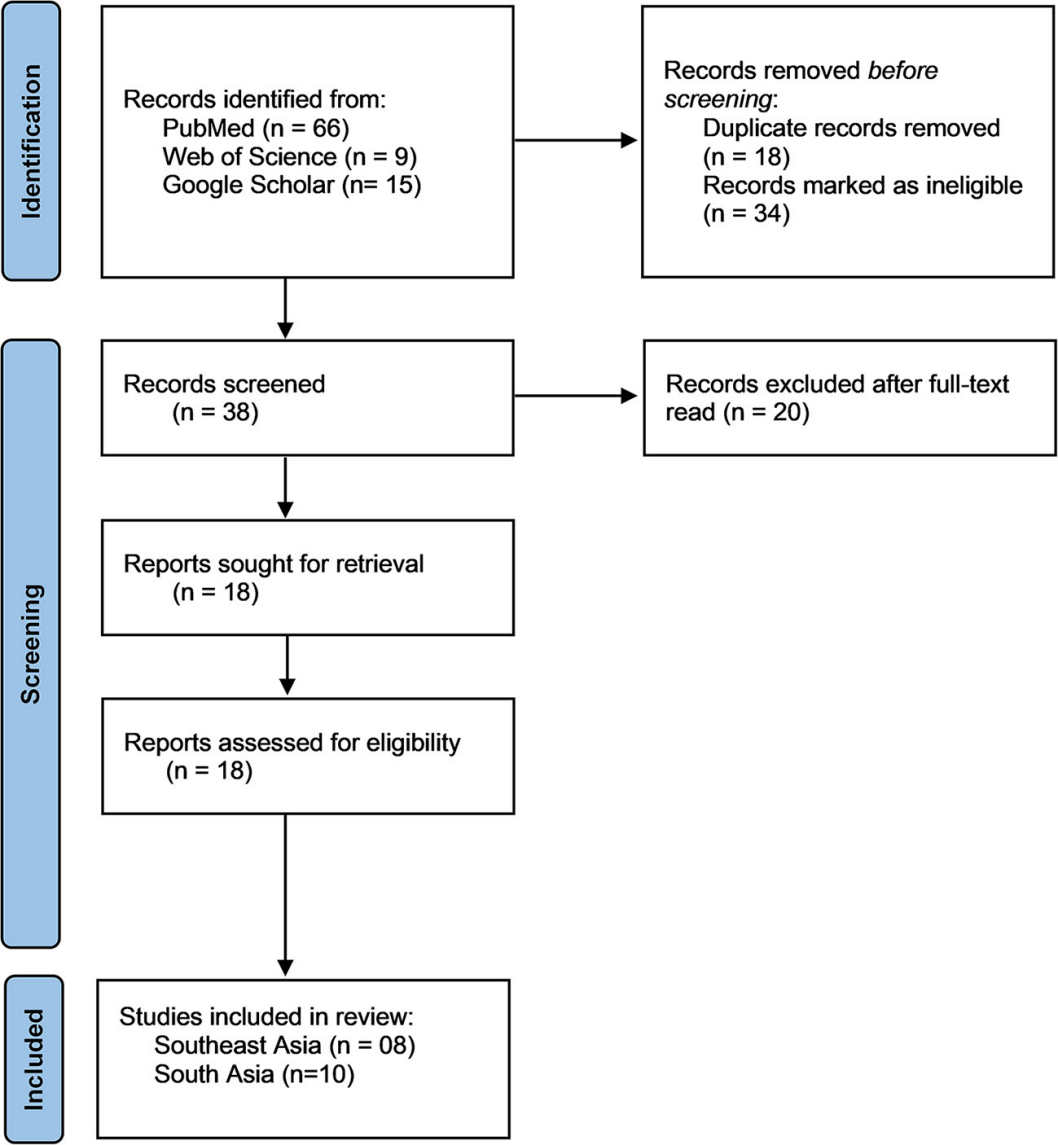
Preferred reporting items for systematic reviews and meta-analyses (PRISMA) 2020 flow diagram for the study selection process.

Articles were selected if they reported the prevalence, mean score and risk factors of knowledge, attitude and practices toward COVID-19, conducted on South and Southeast Asian population, published in peer-reviewed journals in the English language. Studies were excluded if they were not published in indexed journals, case report and editorials. All recorded article was screened by the two independent author MMR and SC, and articles were managed by using Mendeley version 1.19.4 software to exclude duplicates. The steps followed in the literature search are illustrated in Figure 1.

#### Data Screening and Extraction

Following the inclusion and exclusion criteria, excluding duplicate articles, two investigators (MMR and SC) independently assessed the full-text articles for inclusion after screening the titles and abstracts. Any conflict was resolved by consultation with the co-authors. All eligible studies were extracted in a standardize form. For each selected studies, publication details [first author, publication year]; design and population [country, study design, and sample size]; participants' characteristics and major findings [prevalence or proportion, mean/median score of knowledge, attitude, practice with confidence interval, standard deviation, and other related statistics] were extracted.

#### Critical Appraisal and Publication Bias

The critical appraisal of each selected study was assessed by SC and MNH independently using Hoy et al., for-quality assessment criteria (31, 32). The quality of each selected studies was assessed by using a set of nine dichotomous [yes/no] questions. If any selected study satisfied in a question, then it coded 0 for yes otherwise 1 for no. A funnel plot was performed to determine publication bias.

### Statistical Analysis

To analyze cross-sectional data, the participant's responses were extracted from Google form and exported into Microsoft Excel 2010 for necessary data management [checking, cleaning and coding]. Frequency distribution was employed first to understand the participants' demographics characteristics. The prevalence of knowledge, attitude and practices were displayed through a bar diagram with a 95% confidence interval. Significant association of COVID-19 knowledge, attitudes, and practices with participant's socio-demographic profiles were tested by the Pearson chi-square test. The degree of associated risk factors was assessed by an adjusted binary logistic regression model (33, 34). To do this, we considered 5% as the significant level. All data management and statistical analysis were carried out by Microsoft Excel-2010 and IBM SPSS Statistics 20.

To analyze systematic review data, we first extracted information through Microsoft Excel-2010. The descriptive statistics were assessed first and the pooled prevalence of knowledge, attitude and prevention practice were examined using random effect model (35–38). Subgroup analysis for KAP were performed according to region (Southeast and South Asia). Heterogeneity was observed by Cochrane Q and I^2^ statistics (39–42). Finally, we assessed the significant risk factors of person's knowledge, attitude and practice toward COVID-19. For meta-analysis, all data management and statistical analysis were carried out by STATA 17 version.

### Ethics and Permission for Data Collection

Following the standards of Helsinki Declaration and its corresponding modifications or similar ethical principles, this study was carried out. The data was collected through an online survey where written informed consent was taken from each participant. Respondents who expressed their consent, after reading the aforementioned, to take part in the study by clicking either “Yes” or “No” were included in the study. Those who did not consent by clicking “No” were not included in the study. Ethics approval and permission for data collection were granted by the Asia Metropolitan University Medical Research and Ethics Committee with the registration number AMU/FOM/NF/202016.

## Results

### Demographics Characteristics of the Participants

A total of 743 respondents were responded; 53.2% were <25 years old, and 61.5 % were female. Almost three-fourths came from the urban area, and more than half (52.8%) were Malay, followed by Indians (35.5%). Nearly all respondent was of Malaysian nationality. The majority were (73.8%) had a tertiary level of education. More than half of respondents were Employed (50.9%), lived with 5–8 family members (51.8%), and their monthly family income was between 4,850–10,959 RM (42.8%) (Table 1).

**Table 1:** Socio-demographic association of person's knowledge, attitude and practices.

**Socio-demographic factors**			**Knowledge (*****N*** **=** **743)**			**Attitudes (*****N*** **=** **743)**			**Practices (*****N*** **=** **743)**		
		**Total**	**High knowledge** ***n* (%)**	**Low knowledge** ***n* (%)**	***P*-value**	**Positive attitude** **n (%)**	**Negative attitude** **n (%)**		**Good** **n (%)**	**Poor** **n (%)**	***P*-value**
Age	18–24	395 (53.2)	233 (31.4)	265(35.7)	0.0001	277(37.3)	221 (29.7)	0.007	238 (32.0)	260 (35.0)	0.291
	25–44	207 (27.9)	76 (10.2)	50 (6.7)		60 (8.1)	66 (8.9)		70 (9.4)	56 (7.5)	
	>45	141 (19)	82 (11.0)	37 (5.0)		48 (6.5)	71 (9.6)		60 (8.1)	59 (7.9)	
Gender	Male	286 (38.5)	157 (21.1)	129 (17.4)	0.327	133 (17.9)	153 (20.6)	0.022	106 (14.3)	180 (24.2)	.000
	Female	457 (61.5)	234 (31.5)	223 (30.0)		252 (33.9)	205 (27.6)		262 (35.3)	195 (26.2)	
Residence	Rural	180 (24.2)	87 (11.7)	93 (12.5)	0.185	98 (13.2)	82 (11.0)	0.418	81 (10.9)	99 (13.3)	0.163
	Urban	563 (75.8)	304 (40.9)	259 (34.9)		287 (38.6)	276 (37.1)		287 (38.6)	276 (37.1)	
Ethnicity	Malay	392 (52.8)	212 (28.5)	180 (24.2)	0.727	198 (26.6)	194 (26.1)	0.732	170 (22.9)	222 (29.9)	.002
	Chinese	62 (8.3)	31 (4.2)	31 (4.2)		31 (4.2)	31 (4.2)		31 (4.2)	31 (4.2)	
	Indian	264 (35.5)	137 (18.4)	127 (17.1)		141 (19.0)	123 (16.6)		155 (20.9)	109 (14.7)	
	Others	25 (3.4)	11 (1.5)	14 (1.9)		415 (2.0)	10 (1.3)		12 (1.6)	13 (1.7)	
Nationality	Malaysian	726 (97.7)	381 (51.3)	345 (46.4)	0.605	374 (50.3)	352 (47.4)	0.282	359 (48.3)	367 (49.4)	0.776
	Non- Malaysian	17 (2.3)	10 (1.3)	7 (0.9)		11 (1.5)	6 (0.8)		9 (1.2)	8 (1.1)	
Education level	Uneducated	3 (0.4)	2 (0.3)	1 (0.1)	0.0165	2 (0.3)	1 (0.1)	0.828	257 (34.6)	291 (39.2)	
	Primary	1 (0.1)	1 (0.1)	0 (0.0)		1 (0.1)	0 (0.0)		34 (4.6)	32 (4.3)	0.72
	Secondary	35 (4.7)	25 (3.4)	10 (1.3)		17 (2.3)	18 (2.4)		158 (21.3)	154 (20.7)	
	Post-secondary	156 (21)	82 (11.0)	74 (10.0)		83 (11.2)	73 (9.8)		4 (0.5)	2 (0.3)	
	Tertiary education	548 (73.8)	281 (37.8)	267 (35.9)		282 (38.0)	266 (35.8)		172 (23.1)	187 (25.2)	
Occupation	Part time employed	66 (8.9)	36 (4.8)	30 (4.0)	0.14	36 (4.8)	30 (4.0)	0.099	34 (4.6)	32 (4.3)	0.72
	Full time employed	312 (42)	174 (23.4)	138 (18.6)		145 (19.5)	167 (22.5)		158 (21.3)	154 (20.7)	
	Part time student	6 (0.8)	5 (0.7)	1 (0.1)		3 (0.4)	3 (0.4)		4 (0.5)	2 (0.3)	
	Full time student	359 (48.3)	176 (23.7)	183 (24.6)		201 (27.1)	158 (21.3)		172 (23.1)	187 (25.2)	
Marital Status	Single	497 (66.9)	240 (32.3)	257 (34.6)	0.006	270 (36.3)	227 (30.6)	0.10	238 (32.0)	259 (34.9)	0.549
	Married	224 (30.1)	140 (18.8)	84 (11.3)		106 (14.3)	118 (15.9)		120 (16.2)	104 (14.0)	
	Divorced	11 (1.5)	7 (0.9)	4 (0.5)		3 (0.4)	8 (1.1)		6 (0.8)	5 (0.7)	
	Widowed	2 (0.3)	1 (0.1)	1 (0.1)		2 (0.3)	10 (0.0)		1 (0.1)	1 (0.1)	
	Others	9 (1.2)	3 (0.4)	6 (0.8)		4 (0.5)	5 (0.7)		3 (0.4)	6 (0.8)	
No. of family members	<5	311 (41.9)	169 (22.7)	142 (19.1)	0.077	165 (22.2)	146 (19.7)	0.385	163 (21.9)	148 (19.9)	0.402
	5 to 8	385 (51.8)	191 (25.7)	194 (26.1)		192 (25.8)	193 (26.0)		182 (24.5)	203 (27.3)	
	8+	47 (6.3)	31 (4.2)	16 (2.2)		28 (3.8)	19 (2.6)		23 (3.1)	24 (3.2)	
Family income	< RM 4 849	256 (34.5)	139 (18.7)	117 (15.7)	0.639	142 (19.1)	114 (15.3)	0.211	125 (16.8)	131 (17.6)	0.081
	RM 4850 to 10959	318 (42.8)	161 (21.7)	157 (21.1)		164 (22.1)	154 (20.7)		147 (19.8)	171 (23.0)	
	> RM 10 960	169 (22.7)	91 (12.2)	78 (10.5)		79 (10.6)	90 (12.1)		96 (12.9)	73 (9.8)	

### Sources of Information Toward COVID-19 Among Adult Population

**Figure 2:**
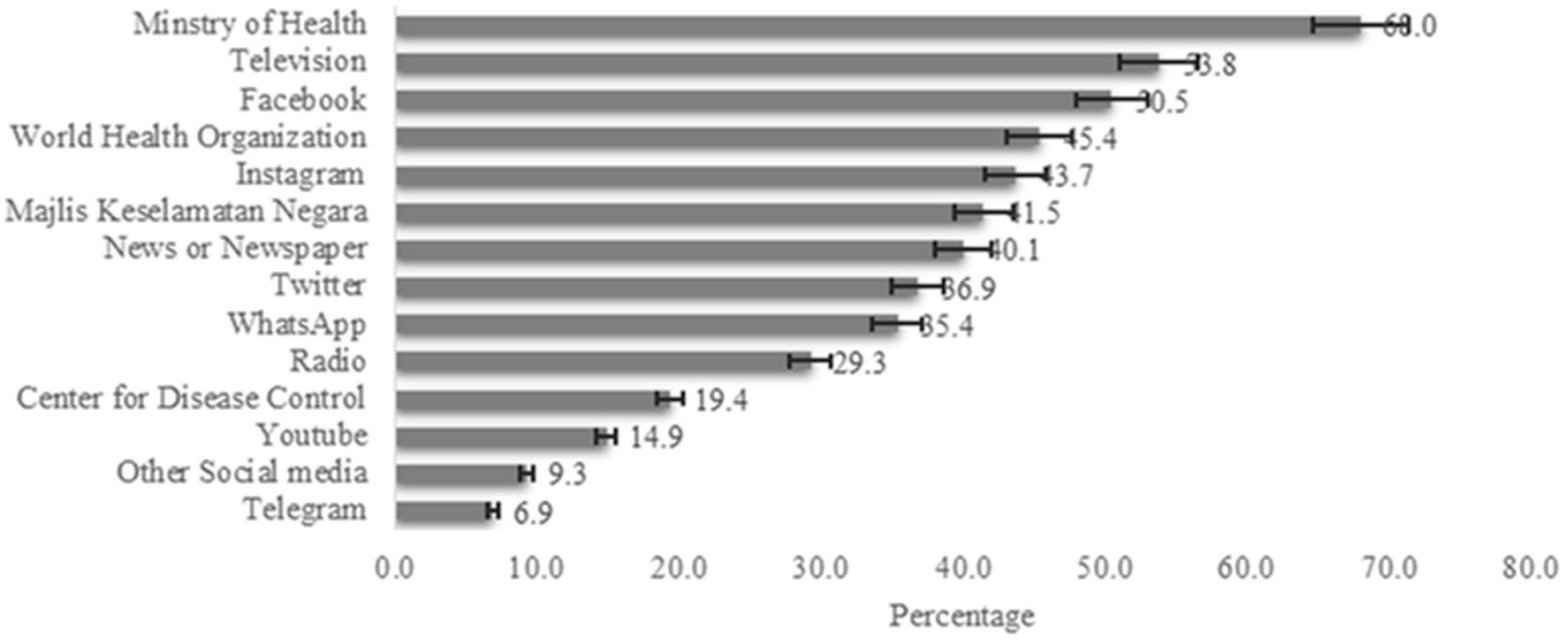
Sources of Information of COVID19 among adult population in Malaysia (*n* = 743).

Figure 2 reported the sources of information of COVID-19 among the general population. Ministry of Health (68%) were the major source of information of COVID-19, followed by Television (53.8%), Facebook (50.5%) and WHO (45.4%).

### Distribution of Knowledge, Attitude and Practice by Age, Sex, Residence and Ethnicity

**Figure 3:**
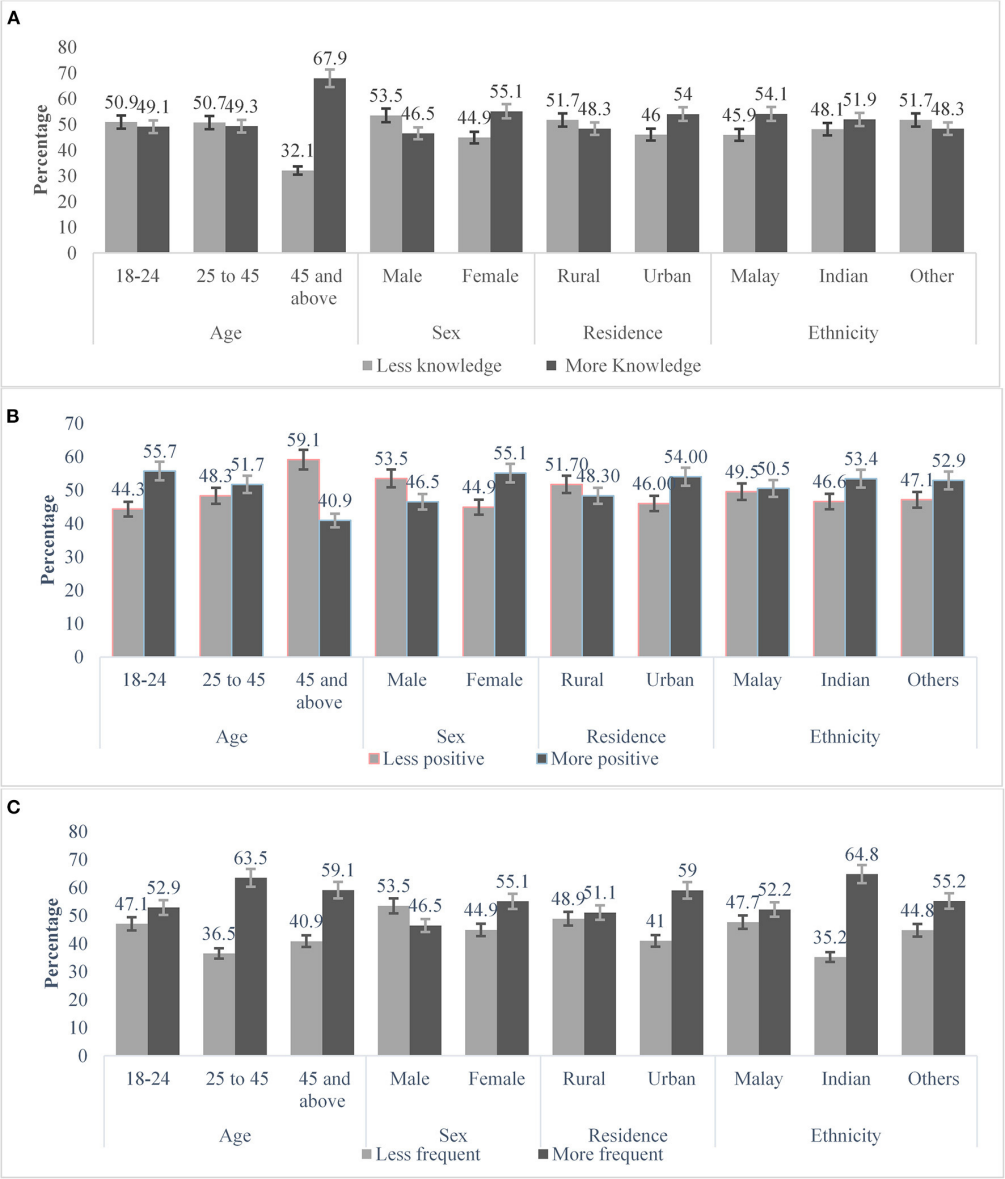
Respondent's knowledge **(A)**, attitude **(B)** and practice **(C)** patterns toward COVID-19. The vertical axis represents percentage and parallel axis are the respondent's age, sex, residence and ethnicity.

Out of 743 respondents included in the cross-sectional study, the prevalence of good knowledge, positive attitude and frequent practice toward COVID-19 epidemic were found 52.6%, 51.8% and 57.1%, respectively. The prevalence of COVID-19 knowledge, attitude and practices score were 52.6%, 51.8% and 57.1% with Mean score 6.54 [SD: 1.38], 32.45 [SD: 4.29] and 32.0 [4.92] respectively. Participant's knowledge toward COVID-19 found to increase with the increase of age, and persons aged 45 or above had more knowledge [67.9%] toward COVID-19. Females had found more knowledge [55.1%] where 46.5% found more knowledge among males. The participant whose ethnicity is Malay [54.1%] and living in urban [54.0%] areas had more knowledge toward COVID-19 (Figure 3A).

The participant's positive attitude toward COVID-19 were found [55.7%] more in the age group 18-24 years and the negative attitude was highest [59.1%] among the people aged 45 and over. Females were showed more positive attitude than males [55.1 vs. 53.5%]. The urban and participants whose ethnicity were Indian responded more positive attitude than others (Figure 3B).

Participant's distribution of practice toward COVID-19 according to their age, sex, residence and ethnicity were presented in Figure 3C. The participants aged 25–45 years were more frequent [63.5%] in practice toward COVID-19 than any other age groups. Female [55.1%], urban [59.0%] and participants whose ethnicity were Indian [64.8%] were found more frequent in practice toward COVID-19 (Figure 3C).

### Socio-Demographic Association of Knowledge, Attitude and Practices Toward COVID-19

The association between participant's socio-demographic factors with knowledge, attitude and practice were summarized in Table 1. The Pearson Chi-square test was performed to examine the significant associated factors of KAP. Respondent's age, education and marital status were significantly [*p* < 0.05] associated with participant's knowledge. Participant's age and gender were significantly [*p* < 0.05] associated with the attitude toward COVID-19. Respondent's sex and ethnicity were also significantly [*p* < 0.05] associated with their practices toward COVID-19 (Table 1).

### Risk Factors Associated With Knowledge, Attitude and Practice Toward COVID-19

Participant's socio-demographic risk factors were identified, observing the degree of association [odds ratio] obtained by the adjusted binary logistic regression model. Adjusted binary logistic regression analysis revealed that the odds of knowledge toward COVID-19 infections were 1.63 times higher among the person aged over 45 than those participants belonging in 18–24 years age group [AOR: 1.63, 95% CI: 0.81–3.27]. The other socio-demographic risk factors of more frequent knowledge were being tertiary education [AOR: 0.52, 95% CI: 0.24–1.13], being non- Malaysian (AOR: 1.34, 95% CI: 0.47–3.80), and ever married [AOR: 1.40, 95% CI: 0.85–2.31]. The participants aged over 45, female, living in urban areas, Indian ethnic, post-secondary educated, and had monthly > RM 10,960 income were more likely to practice toward COVID-19 (Table 2).

**Table 2:** Risk factors associated with Knowledge, attitude and practice for COVID-19 among general population.

**Characteristics**		**Knowledge**	**Attitude**	**Practices**
		**AOR (95% C.I.)**	**AOR**	**AOR**
Age	18 to 24	Ref	Ref	Ref
	25 to 45	0.92 (0.56–1.51)	0.72 (0.43–1.25)	1.9 (1.09–3.31)
	>45	1.63 (0.81–3.27)	0.49 (0.24–1.01)	2.01 (0.93–4.31)
Gender	Male	Ref	Ref	Ref
	Female	0.82 (0.58–1.14)	0.88 (0.62–1.24)	3.02 (2.13–4.29)
Place	Rural	Ref	Ref	Ref
	Urban	1.31 (0.91–1.89)	0.80 (0.55–1.18)	1.30 (0.87–1.94)
Ethnicity	Malay	Ref	Ref	Ref
	Indian	0.81 (0.57–1.15)	0.79 (0.55–1.15)	2.22 (1.51–3.26)
	Other	0.75 (0.45–1.23)	0.76 (0.45–1.29)	1.08 (0.63–1.85)
Nationality	Malaysian	Ref	Ref	Ref
	Non–Malaysian	1.34 (0.47–3.80)	1.94 (0.63–5.92)	1.99 (0.55–7.24)
Education	School Education	Ref	Ref	Ref
	Post–Secondary Education	0.56 (0.25–1.28)	0.75 (0.33–1.70)	1.58 (0.68–3.65)
	Tertiary education	0.52 (0.24–1.13)	0.89 (0.42–1.93)	0.91 (0.42–1.98)
Occupation	Employed	Ref	Ref	Ref
	Students	1.25 (0.78–1.99)	1.13 (0.69–1.85)	1.03 (0.61–1.72)
Marital status	Single	Ref	Ref	Ref
	Ever married	1.40 (0.85–2.31)	1.15 (0.68–1.93)	0.88 (0.51–1.53)
Family member	<5	Ref	Ref	Ref
	5 to 8	0.88 (0.63–1.23)	0.84 (0.59–1.19)	1.19 (0.83–1.71)
	>8	1.63 (0.82–3.22)	1.31 (0.66–2.62)	1.09 (0.53–2.28)
Monthly Income	< RM4,849	Ref	Ref	Ref
	RM4,850 to RM10,959	0.84 (0.59–1.19)	0.99 (0.69–1.43)	0.84 (0.57–1.22)
	> RM 10,960	0.73 (0.47–1.13)	0.70 (0.45–1.11)	1.87 (1.14–3.03)

### Degree of Practices Based on Participant's Knowledge and Attitude

Participant's degree of practices was determined based on their knowledge and attitude obtained by adjusted binary logistic regression model (Table 3). It was investigated that the participants who were belonging to more knowledge were significantly more [AOR: 1.50; 95% CI: 1.11–2.03] likely to practice toward COVID-19. Again, the participants who were more positive in attitude, were significantly 3.03 [AOR: 2.03; 95% CI: 2.24–4.10] times more likely to practice toward COVID-19.

**Table 3:** Degree of practices based on the participant's knowledge and attitude.

**Characteristics**		**AOR (95% CI)**
Knowledge	Less knowledge	Reference category
	More knowledge	1.50 (1.11–2.03)
Attitude	Less positive	Reference category
	More positive	3.03 (2.24–4.10)

### Systematic Review and Meta-Analysis Results

#### Electronic Search and Study Selection

Initially, our electronic search retrieved 90 articles from three databases and search engine [PubMed, Web of Science and Google Scholar]. After removing duplicate and ineligible articles, 38 articles remained for full text screening. Out of 38 articles full-text screened, 20 were removed due to not satisfying inclusion and exclusion criteria. Therefore, 18 articles from Southeast Asia [*n* = 08] and South Asia [*n* = 10] were included in this study to assess and analysis (Figure 1).

#### Study Risk of Bias Assessment

The risk of bias score was evaluated for each of the studies using the guideline described by Hoy et al. in the year 2012 and are summarized in Supplementary Table S2. For each selected studies, the assessment score was ranged from 0–9. Studies that scored 0–3 were considered as having low risk of bias, and studies with scores 4–6 were moderate risk, and studies with scores of 7–9 were considered as having high risk of bias. Out of selected 18 studies, no studies were found as high risk of bias. Twelve studies found low risk of bias and the rest were moderate risk of bias.

#### Prevalence of Knowledge, Attitude and Practice Behaviors in Southeast and South Asia Obtained by Systematic Review and Meta-Analysis

**Figure 4:**
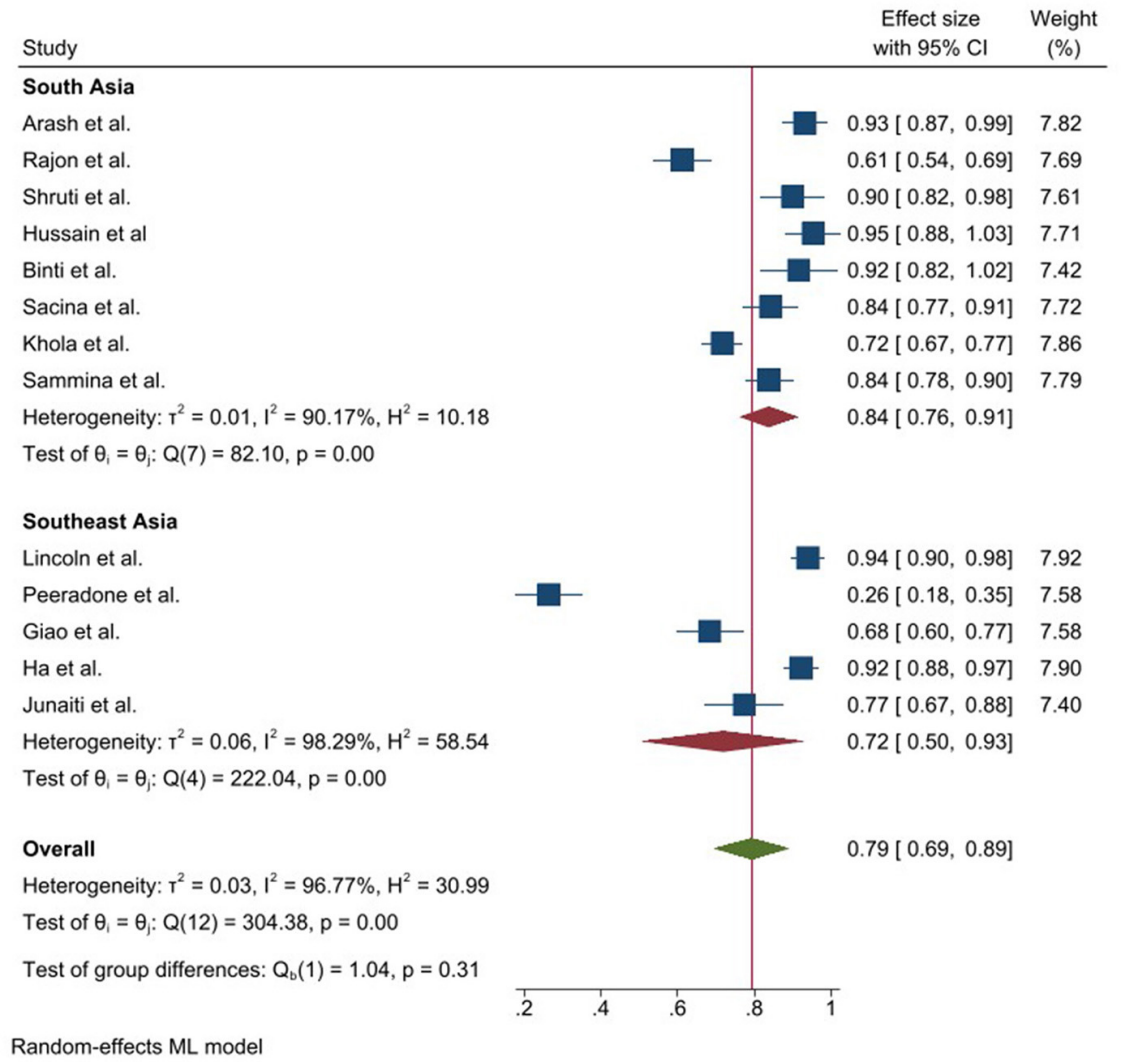
Prevalence of good knowledge toward COVID-19 epidemic in Southeast and South Asia, stratified by region.

Our meta-analysis included 18 articles from 11 countries of Southeast and South-Asia. Most of the studies were conducted in Nepal (*n* = 3) and only one study found in Afghanistan. All the selected studies were conducted within the year of 2020. The minimum sample size was 368, conducted in Indonesia and maximum sample size was 4,850 in Malaysia. Among the selected 18 studies, 13 reported prevalence of knowledge and five reported a mean score of knowledge toward COVID-19. The prevalence of knowledge toward COVID-19 were ranged 26.53% (minimum: Thailand) to 95.4% (maximum: Nepal). However, almost all studies reported more than 50% knowledge prevalence rate in COVID-19 (Table 4). Overall, the pooled prevalence of good knowledge toward COVID-19 was 0.79 (95% CI: 0.69–0.89) whereas the value was 0.84 in South Asia and 0.72 (95% CI: 0.50–0.93) in Southeast Asia respectively, obtained by subgroup analysis. The heterogeneity was 96.77% and the prevalence difference between Southeast and South Asia was insignificant (*p* = 0.31) (Figure 4).

**Table 4:** Characteristics of studies included in the systematic review of knowledge, attitude and practice toward COVID−19 in Southeast and South Asia.

**Author**	**Country**	**Sample**	**Time**	**Significant factor**	**Knowledge (%)**	**Attitude (%)**	**Practice (%)**
Nemat et al. (43)	Afghanistan	1169	Oct. 2020	———-	93.2%	91.1%	97%
Banik et al. (28)	Bangladesh	707	May.2020	Gender, education, place of residence	61.2%	89.0%	51.6%
Shukla et al. (29)	India	570	July, 2020	—-	90.0%	80.0%	90.0%
Hussain et al. (44)	Nepal	760	April, 2020	Gender, occupation	95.4%	78.4%	94.9%
Vaidya et al. (45)	Nepal	380	April, 2020	—-	91.6%	71.5%	94.7%
Paudel et al. (46)	Nepal	766	Mar.–Apr. 2020	Age, marital status, gender, education, occupation, province of residence	84.3%	71.5%	93.1%
Noreen et al. (47)	Pakistan	1474	June, 2020	Gender, education	71.7%	92.5%	95.4%
Mahmood et al. (48)	Pakistan	1000		Gender, education, income	83.9%		65.6%
Lau et al. (23)	Philippine	2224	Feb–Mar., 2020	Place of residence, education	94.0%	82.2%	89.9%
Srichan et al. (49)	Thailand	520	Feb, 2020	Gender, age, education	26.53%	71.5%	90.0%
Huynh et al. (50)	Vietnam	522	Feb.–Mar. 2020	Gender, knowledge level, education, age	68.4%	90.8%	77.2%
Nhu et al. (33)	Vietnam	1999	April, 2020	Age, Sex, marital status, fear	92.2%	68.6%	75.8%
Azlan et al. (1)	Malaysia	4850	Mar.–Apr.2020	Gender, age, region, occupation, income	10.5 ± 1.4 (13)	83.1%	83.4%
Mehmet et al. (51)	Turkey, Malaysia	1320	April, 2020	Gender, education, age, marital status	8.15 ± 1.6 9.99 ± 1.8	59.3%, 79.6%	50.2%, 94.1%
Sahar et al. (52)	Indonesia	368	June, 2020	Source of information, gender, working status	77.4%	33.0 ± 2.7	84.2%
Hossain et al. (53)	Bangladesh	2157	Apr.–May.2020	Age, education, place of residence	8.71 ±1.64 (12)	8.9 ± 1.2 (12)	8.7 ± 1.6
Vyas et al. (54)	India	1231	Apr.–May,2020	Gender, occupation	10.19 ± 1.6 (12)	2.33 ± 0.66	1.97 ± 0.16
Muslih et al. (55)	Indonesia	6249	Apr–May,2020	Gender, place of residence, occupation, major of education	13.14 ± 2.76 (18)	16.56 ± 1.72	31.06 ± 3.80

**Figure 5:**
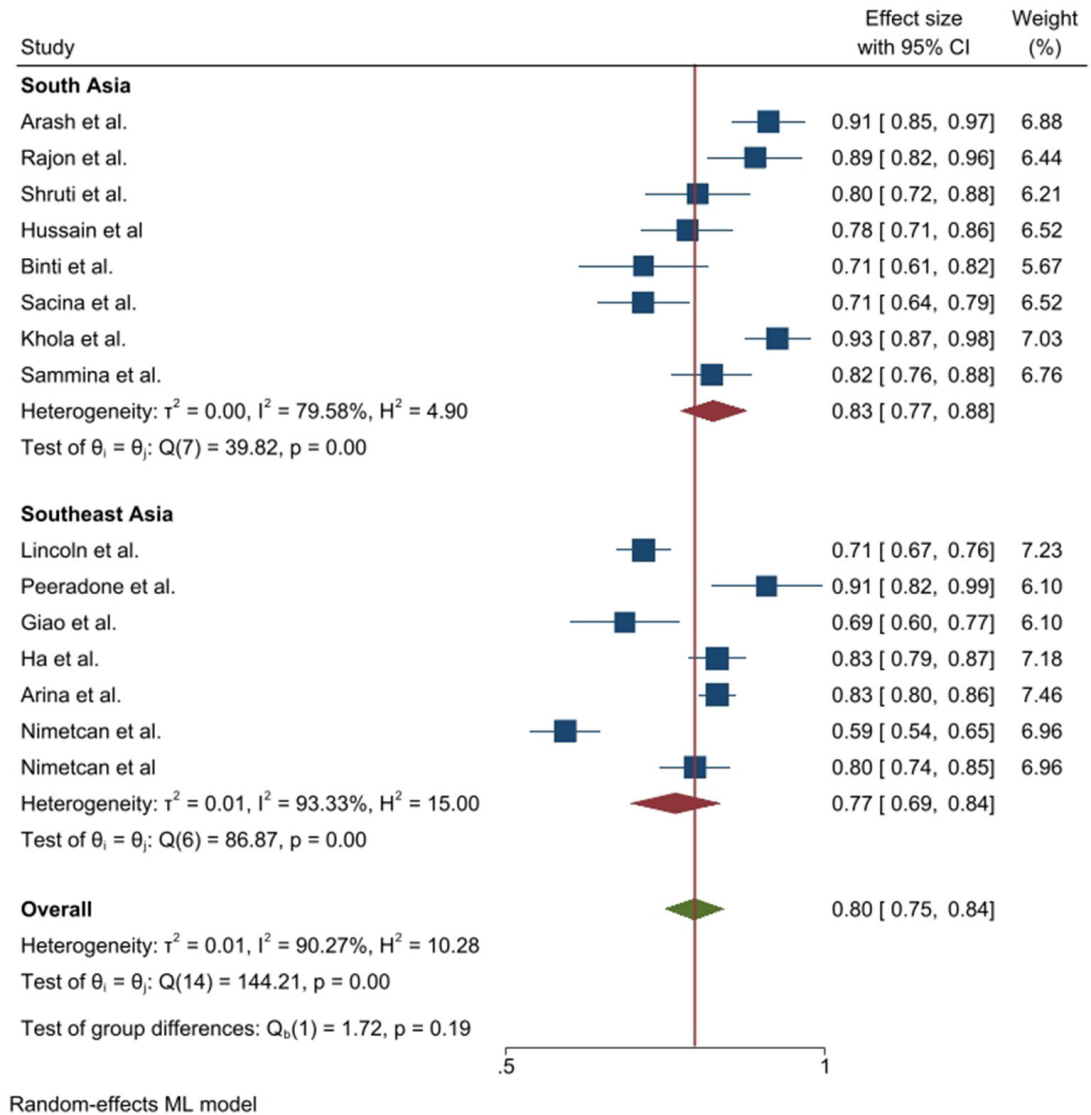
Prevalence of positive attitude toward COVID-19 epidemic in Southeast and South Asia, stratified by region.

The attitude toward COVID-19 was also found satisfactory in South and Southeast Asia. Similar to knowledge, the attitude prevalence was also found more than 50% in all the selected studies where the maximum positive attitude showed in 92.5% in Pakistan and the minimum was in Turkey (59.3%). The pooled prevalence of positive attitude was 0.80 (95% CI: 0.75–0.84) and this value was higher (0.83 vs. 0.77) in South Asia compared to Southeast Asia. Heterogeneity was 90.27% among the study and the prevalence difference between Southeast and South Asia was insignificant (*p* = 0.19) (Figure 5).

**Figure 6:**
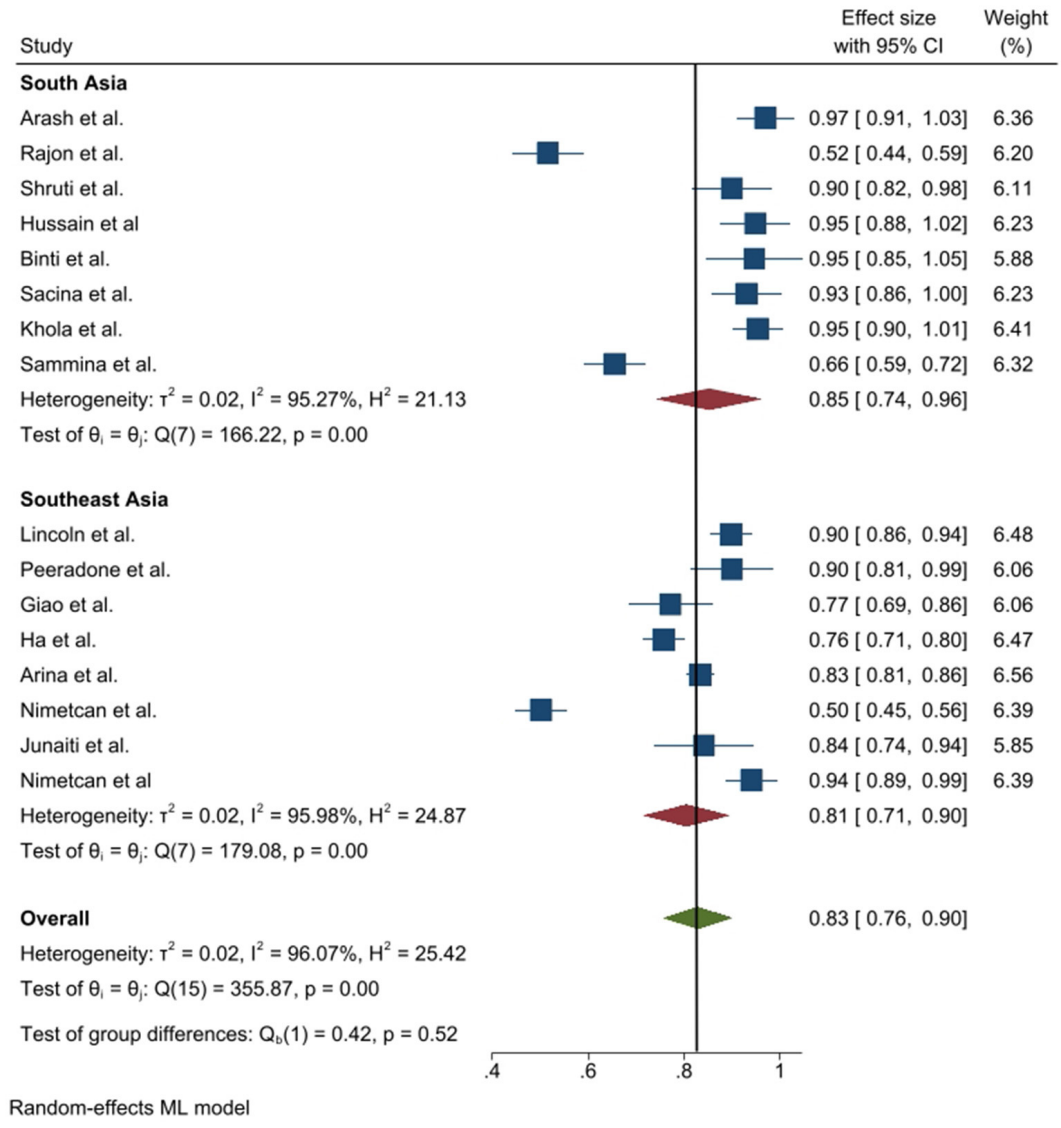
Prevalence of frequent practice toward COVID-19 epidemic in Southeast and South Asia, stratified by region.

In the case of COVID-19 prevention practice, the prevalence was also satisfactory. The maximum prevalence toward COVID-19 prevention practice was found at 97% in Afghanistan where the minimum value was 50.2% in Turkey (Table 4). According to random effect model, the pooled prevalence of frequent practice toward COVID-19 epidemic was 0.83 where this practice was insignificantly different between South Asia and Southeast Asia (0.85 vs. 0.83; *p* = 0.52), obtained by subgroup analysis. The heterogeneity was 96.98% among the studies (Figure 6).

Five studies reported mean scores instead of the prevalence knowledge toward COVID-19, where the mean value ranged from 8.15 ± 1.6 (minimum: Turkey) to 13.14 ± 2.76 (maximum: Indonesia). Four studies reported mean attitude score instead of prevalence and the mean value ranged between 2.33 ± 0.66 (minimum: India) and 33.0 ± 2.7 (maximum: Indonesia). Only three studies reported a mean score of prevention practice toward COVID-19, where the minimum score was reported in India (1.97 ± 0.16) and the maximum was in Indonesia (31.03 ± 3.80).

#### Factors Associated With COVID-19 Patient's Knowledge, Attitude and Practice Obtained by Systematic Review

In our selected studies, almost all authors reported risk factors of knowledge, attitude and practice toward COVID-19. The maximum number of risk factors were observed in the study from Nepal, Malaysia and Turkey, whereas the most frequent risk factors were participant's age, gender, education, place of residence and occupation (Table 4).

#### Publication Bias

This study performed funnel plots to assess publication bias of the prevalence of KAP which is presented in Supplementary Figures S3–S5. Publication bias was present in all knowledge, attitude and practice behaviors.

## Discussion

The present mixed study designed research aimed to assess knowledge on the KAP and their associated risk factors about COVID-19 in Southeast and South Asia. In cross-sectional part of the study, 743 sample was collected from Malaysia through online survey and their socio-demographic and KAP related information was investigated through the univariate and multivariate approaches. In systematic review and meta-analysis part, relevant studies were searched, screened and 18 related studies were meta-analyzed.

According to the cross-sectional findings, the Ministry of Health was the most preferred source of information for COVID-19, followed by television, Facebook, and WHO, respectively. The Malaysian government also created a Telegram channel that was approved by the “Ministry of Health Malaysia and Malaysia Communications and Multimedia Commission (MCMC)”. This attempt by the government to monitor the dissemination of false information about COVID-19 could explain why the Ministry of Health was the most favored source of information (1, 51). In response to COVID-19, different countries in Southeast and South Asia were also raised an alert and implemented wide-ranging, multi-agency public health measures under WHO guidelines to fight against the pandemic. As COVID-19 appears to be transmitted from person to person, almost all countries in Southeast and South Asia initiated a public campaign highlighting the necessity of practicing respiratory hygiene, hand hygiene and using appropriate personal protective equipment (PPE) (1, 23, 28, 29, 43, 44, 47, 49, 55).

Despite the fact that the majority of our cross-sectional sample participants had completed their tertiary education and it has been over a year since COVID-19 was first introduced in Malaysia, our survey showed that more than half of the population had good knowledge level toward COVID-19. The finding is lower than that of our meta-analysis and a previous study conducted in Malaysia (1). A study conducted in Nigeria showed good knowledge level among the participants. Some related survey conducted in Qatar and Iran also revealed high prevalence of having good knowledge toward COVID-19 (56, 57). While, a low percentage of Bangladeshis, Indians and Thailand population had good knowledge (49, 54, 58).

The study found that individuals aged 45+ years had more likely to have COVID-19 knowledge than the younger persons. A study conducted in Saudi Arabia also showed that older individuals had higher knowledge about COVID-19 (59). According to the World Health Organization (WHO) and some existing literatures, older society is at highest risk of contracting this virus, and more than 95% of the related death is attributed to this age group (60–62). This may be because older people are more likely to catch viruses or diseases such as COVID-19 due to poor immunity, so they could be more cautious about COVID-19. In addition, our study showed that non-Malaysian and married citizens have more knowledge than their counterparts. Similar research carried out in Malaysia also showed that knowledge was significantly related to age, and job status (1). Our findings of knowledge are also comparable to other studies conducted in Pakistan and China, in which knowledge score was significantly different among age groups, marital status, level of education and employment status (48, 63, 64).

As far as attitudes are concerned, almost half of the participants demonstrated a positive attitude toward coronavirus in our cross-sectional research and the level was high in Southeast and South Asia obtained by meta-analysis. People aged 25-45, women, urban people, Indian ethnicity, tertiary education, and monthly income between RM 4, 850, and 10,959 are less likely to have a positive attitude than their counterparts and the finding are comparable to a study (21). The WHO South-East Asia region country profile and the IEDCR COVID-19 update state that the number of deaths is higher among elderly persons, males, and those with pre-existing co-morbidities in Bangladesh (65, 66). Non-Malaysian, students, married and having >8 family members were more likely to have a positive attitude against COVID-19. In the previous Malaysian survey, similar results were found that 83% of participants had favorable attitudes toward the regulation of COVID-19 and the attitudes markedly correlated with age, religion and profession (1). However, some studies showed negative emotions like panic and anxiety during a pandemic that could influence their attitudes (5, 67, 68). One study in China showed fear related to age concerning knowledge and occupation, while another study completed in India reported that 80% of people in need of mental health care for COVID-19 experienced fear, anxiety, and depression (69, 70). Other South or Southeast countries, such as Bangladesh, Myanmar and Malaysia also reported high level of psychological distress in general populations (67, 71–74).

The WHO and its member states are emphasizing on the general public to practice COVID-19 preventive measures and our study found more than half of the participants showed good practice level toward COVID-19. Study also highlighted that person aged 45+ years, female, urban residents, Indian ethnicity, non-Malaysian, higher education, ever married and good income were more likely to have good practice and all are significant to COVID-19 prevention practices in Malaysia. By systematic review, we also observed age, gender, region and occupation as the significant factor of persons' practice toward COVID-19 (1, 23, 29, 33, 44–55, 65). Hence, these findings suggest that health education interventions targeting particular groups such as younger people, men, lower educated persons, people with a less monthly income and rural residents should be designed in order to improve COVID-19 awareness, which can play vital role in improving the practice of individual's preventive attitudes (1, 64). It is a common consensus that a more educated population about any given disease will comply better with the preventive and treatment measures (21, 63). Our study also discovered that people with good knowledge and positive attitude were likely to have more prevention practice level toward COVID-19. Similar findings were observed in a KAP survey for COVID-19 (23, 29, 43). Women are more likely to wear a mask when leaving home compared to men (55, 63). Our finding is consistent with a study conducted in China that found a significant association between men and potentially lower practices toward COVID-19, including going to a crowded place during the pandemic or not wearing a mask when leaving home (75).

In our study, the prevalence of KAP found higher in South Asia compared to Southeast Asia. Therefore, Southeast Asian population need more knowledge and awareness programs regarding COVID-19. Although heterogeneity and bias were observed among the study, our cross-sectional study and meta-analysis found lower age group, male, rural residents, illiterate and low-income holder as the vulnerable as the vulnerable to KAP toward COVID-19.

This study offers several implications for the practitioners, governments and health agencies not only regarding the COVID-19 but also for the upcoming disease. Firstly, this study helps countries to know about individuals KAP level; it can assist them to design different awareness programs. Besides, it also helps which group of the individuals requires more attention, such as people less than 24 years, male, living in rural areas, single, and less family income. Secondly, the present study reveals that government not only have to design the awareness but also control mechanism must be developed for monitoring perspective. In addition, non-governmental agencies must participate in the awareness program since people living in rural areas have poor knowledge, attitude and adopted practices. Finally, the government must advised strictly prohibit people not to shake hands, avoid hugs and keep social distancing.

There are some flaws in our research. Firstly, in cross-sectional part, individuals who did not have access to the internet were not able to participate particularly the elderly and people who reside in remote areas. Consequently, results could not apply to the whole population. Secondly, since it was an online survey, there was a possibility of reporting bias. In systematic review part, we found some study reported prevalence of KAP and some were reported mean/median value of KAP which created different dimension in result interpretation. However, we conclude that our survey provides valuable insight into Southeast and South-Asian viewpoints, and the effect of the COVID-19 pandemic on these regions. Therefore, the results can be used by health officials to develop outreach programs and health strategies.

## Conclusions

Overall, the prevalence of high knowledge, positive attitude and good practice toward COVID-19 were found 26.53–95.4%, 59.3%-92.5%, and 50.2%−97.0%, respectively among the Southeast and South Asian population. The cross-sectional study showed that having more knowledge and attitude were encouraging more likely to practice toward COVID-19. Higher aged people, female, urban residents, Indian ethnic and tertiary education are significant factors to KAP toward COVID-19 in Southeast and South Asia. The systematic review results can be used to inform that how others countries in south Asia and south-east Asia enrich their knowledge during the pandemic. The findings of this mixed study conducted on separate background respondents may be helpful for health professionals and policymakers to develop targeted interventions and effective practices. A comprehensive awareness-raising program through mass media as well as the internet and social media is required in parallel with government awareness program for the general people to learn and understand the seriousness of the outbreak.

## Data Availability Statement

The cross-sectional datasets generated and/or analyzed during the current study are available from the corresponding author on reasonable request to meshbah037@gmail.com. The systematic review datasets included in this study are provided in the paper (Table 4). No additional supporting data are available.

## Ethics Statement

The studies involving human participants were reviewed and approved by Asia Metropolitan University Medical Research and Ethics Committee with the registration number AMU/FOM/NF/202016. The patients/participants provided their written informed consent to participate in this study.

## Author Contributions

MR: conceptualization, data curation, formal analysis, investigation, methodology, resources, software, supervision, validation, visualization, writing-original draft, and writing-review and editing. RM: conceptualization, data curation, formal analysis, investigation, methodology, supervision, validation, project administration, resources, visualization, writing-original draft, and writing-review and editing. MH: visualization, writing- original draft, and writing-review and editing. SC and GP: data curation, writing-original draft, and writing-review and editing. KA, WS, AM, DC, SK, AF, FS, and SQ: investigation, project administration, resources, visualization, writing-original draft, and writing-review and editing. YL: funding acquisition, writing-original draft, and writing-review and editing. All authors contributed to the article and approved the submitted version.

## Funding

This work was supported by the Special Projects of the Central Government Guiding Local Science and Technology Development, China [No.2021L3018]. The funder was not involved in study design, in the collection, analysis and interpretation of data; in the writing of the manuscript; nor in the decision to submit the manuscript for publication.

## Conflict of Interest

The authors declare that the research was conducted in the absence of any commercial or financial relationships that could be construed as a potential conflict of interest.

## Publisher's Note

All claims expressed in this article are solely those of the authors and do not necessarily represent those of their affiliated organizations, or those of the publisher, the editors and the reviewers. Any product that may be evaluated in this article, or claim that may be made by its manufacturer, is not guaranteed or endorsed by the publisher.
