# Supplementary material for: Knowledge, Attitude and Practices Toward Coronavirus Disease (COVID- 19) in Southeast and South Asia: A Mixed Study Design Approach
Source: Front Public Health. 2022 Jun 21;10:875727. doi: 10.3389/fpubh.2022.875727 (PMC9253590; doi:10.3389/fpubh.2022.875727)
Supplement: Supplementary file 1 [file Data_Sheet_1.docx]

Supplementary Material

# Supplementary Figures and Tables

**Supplementary 2 Table.** Quality assessment of selected studies (Q1, Q2, Q3……Q10 denotes ten parameters described by Hoy et al for-quality assessment)

| **Authors** | Q1 | Q2 | Q3 | Q4 | Q5 | Q6 | Q7 | Q8 | Q9 | Total score | Quality |
| --- | --- | --- | --- | --- | --- | --- | --- | --- | --- | --- | --- |
| Arash et al.,[24] | 0 | 1 | 1 | 0 | 0 | 0 | 0 | 0 | 0 | 2 | Low |
| Rajon et al.,[25] | 0 | 1 | 1 | 0 | 0 | 0 | 1 | 1 | 0 | 4 | Moderate |
| Shukla et al.,[26] | 0 | 1 | 0 | 0 | 0 | 0 | 1 | 0 | 0 | 2 | Low |
| Hussain et al., [27] | 1 | 1 | 1 | 0 | 0 | 0 | 1 | 0 | 0 | 4 | Moderate |
| Binit et al., [28] | 0 | 0 | 0 | 0 | 0 | 0 | 0 | 1 | 0 | 1 | Low |
| Sachina et al.,[29] | 0 | 0 | 1 | 0 | 0 | 0 | 1 | 0 | 0 | 2 | Low |
| Khola et al.,[30] | 0 | 0 | 0 | 0 | 0 | 0 | 0 | 1 | 0 | 1 | Low |
| Sammina et al., [31] | 0 | 0 | 1 | 0 | 0 | 0 | 0 | 1 | 0 | 2 | Low |
| Lincoln et al.,[14] | 0 | 0 | 0 | 0 | 0 | 0 | 1 | 0 | 0 | 1 | Low |
| Peeradone et al.,[32] | 0 | 0 | 0 | 0 | 0 | 0 | 0 | 0 | 0 | 0 | Low |
| Giao et al.,[24] | 0 | 1 | 0 | 0 | 0 | 0 | 1 | 1 | 0 | 3 | Low |
| Ha et al.,[33] | 1 | 1 | 1 | 0 | 0 | 0 | 1 | 0 | 0 | 4 | Moderate |
| Arina et al., [1] | 0 | 0 | 0 | 0 | 0 | 0 | 1 | 0 | 0 | 1 | Low |
| Nimetcan et al.,[34] | 1 | 1 | 1 | 0 | 0 | 0 | 1 | 0 | 0 | 4 | Moderate |
| Junaiti et al.,[35] | 0 | 1 | 1 | 0 | 0 | 0 | 1 | 0 | 0 | 3 | Low |
| Mohammad et al.,[36] | 0 | 1 | 1 | 0 | 0 | 0 | 1 | 0 | 0 | 3 | Low |
| Himali et al.,[37] | 1 | 1 | 1 | 0 | 0 | 0 | 1 | 1 | 0 | 5 | Moderate |
| Muhammad et al.,[38] | 1 | 1 | 1 | 0 | 1 | 0 | 0 | 0 | 0 | 4 | Moderate |


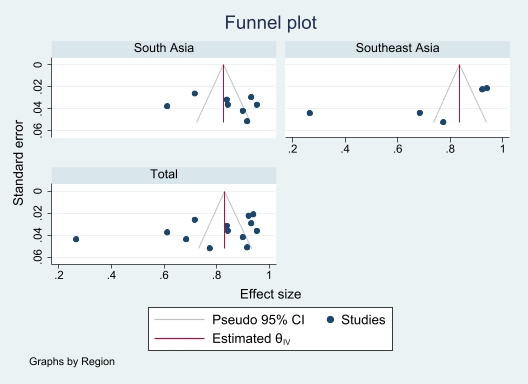


**Supplementary Figure 3.** Funnel plot of the prevalence of good knowledge towards COVID-19 epidemic in South Asia and Southeast Asia.

**
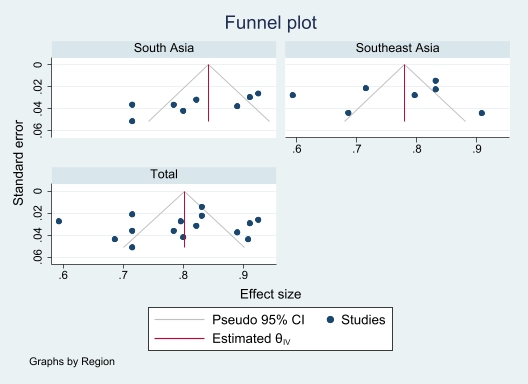
**

**Supplementary Figure 4.** Funnel plot of the prevalence of positive attitude towards COVID-19 epidemic in South Asia and Southeast Asia.

**
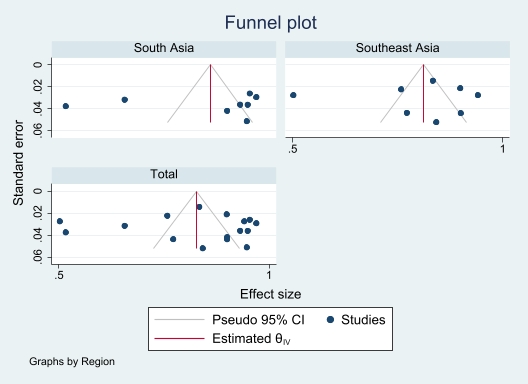
**

**Supplementary Figure 5.** Funnel plot of the prevalence of frequent practice towards COVID-19 epidemic in South Asia and Southeast Asia.
